# Supplementary figures and images for: Mean platelet volume and mean platelet volume to platelet count ratio as predictors of severity and mortality in sepsis
Source: PLoS One. 2022 Jan 6;17(1):e0262356. doi: 10.1371/journal.pone.0262356 (PMC8735631; doi:10.1371/journal.pone.0262356)

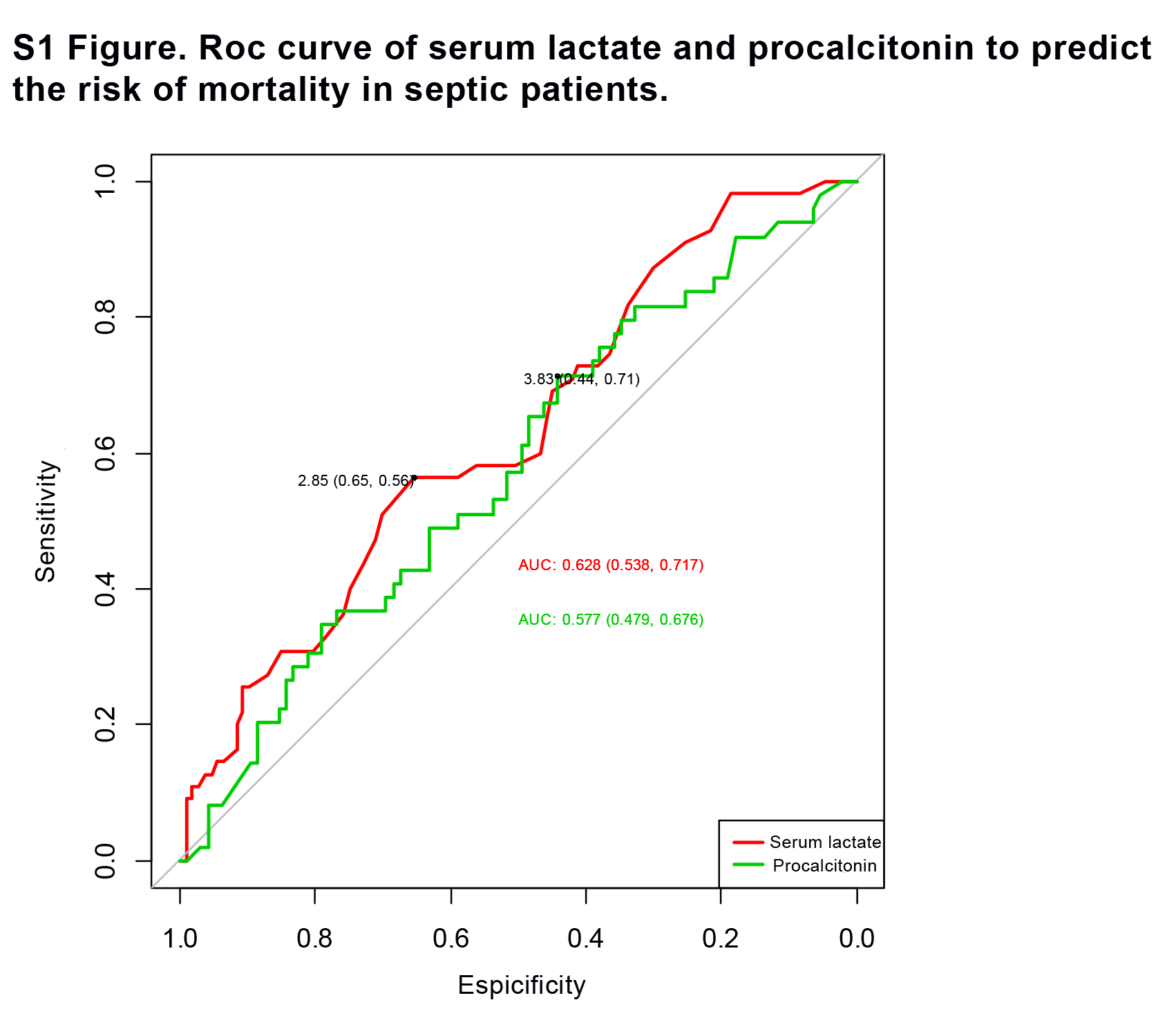

Supplement: S1 Fig — Serum lactate and procalcitonin Roc curve to predict the risk of mortality in septic patients. (TIF) [file pone.0262356.s001.tif]
