# Supplementary material for: Mean platelet volume and mean platelet volume to platelet count ratio as predictors of severity and mortality in sepsis
Source: PLoS One. 2022 Jan 6;17(1):e0262356. doi: 10.1371/journal.pone.0262356 (PMC8735631; doi:10.1371/journal.pone.0262356)
Supplement: Attachment — Submitted filename: Response to Reviewers.docx [file pone.0262356.s002.docx]

**Review Comments to the Author**

**Reviewer #1:** The aim of the authors is to assess the value of MPV and MPV to platelet count ratio as predictors of severity and mortality in patients affected by sepsis. This has been previously done in various series of patients and clinical settings, and in sepsis in particular. Nevertheless, the manuscript may be of clinical interest if properly revised in order to highlight potential points of interest.

**Response:**

Dear reviewer thank you for your comment and for allowing us to review the manuscript. We agree with you, some studies evaluate the behavior of the mean platelet volume in several clinical scenarios and sepsis in particular. However, this study concerns interest, since it was carried out in Quito, a high-altitude city (2850 MASL), where the hypobaric hypoxia increases platelet reactivity. This could induce a prothrombotic phenotype determining an unusual behavior of the platelet indices. (Rocke AS, Paterson GG, Barber MT, Jackson AIR, Main S, Stannett C, et al. Thromboelastometry and platelet function during acclimatization to high altitudes. Thrombi Haemost. 2018; 118 (1): 63–71).

In particular, I would to raise some major issues concerning the content of the manuscript:

- the authors consider sepsis as all the same event, without giving detail whether it is of bacteric vs. viral origin, nor describe in detail the infective agents. A stratification of patients based on the nature of the infection and assessment of potential difference in influencing MPV and MPV to platelet count ratio may increase the value of the works.

**Response:**

The study was carried out only in patients with sepsis of bacterial etiology, with diverse infectious foci such as pulmonary, abdominal, urinary, and others; the site of infection was not associated with mortality in the bivariate statistical analysis, so we did not perform subgroup analysis. This was stated and clarified in the manuscript (lines 82, 120-127). Unfortunately, we did not have access to the data to describe in detail the infective agents; this is a limitation of our study.

- the authors consider only the first 3 days for MPV monitoring (as the most published reports do). Extending the period of observation to the whole period of the hospitalization of the patients, assessing the dynamics, and tracing the trajectory of MPV may also be very useful in understanding this condition and constitute the very point of novelty of the works.

**Response:**

The kinetics of MPV in sepsis parallels the response to the antibiotic treatment administered; that is, it tends to decrease in patients in whom the systemic infection has been controlled and this is observed between 48 to 72 hours of management. For this reason, follow-up during the initial 72 hours is important (lines 249-252). Most studies on this subject, like ours, measure the MPV in the first hours after admission. However, we agree with you, few studies followed up to 15 days after the patients were discharged from intensive care and evaluated 90-day mortality. For example, Vardon Bounes F, Gratacap MP, Groyer S, et al. Kinetics of mean platelet volume predicts mortality in patients with septic shock. PLoS One. 2019; 14 (10): e0223553. Published 2019 Oct 17. doi: 10.1371 / journal.pone.0223553; that found that patients with MPV > 11.6 fL at day 10 of follow-up did not survive (HR: 3.79). This highlights the usefulness of this biomarker both in the critical unit and in hospitalization to determine the prediction of important outcomes such as mortality and prompts us to carry out longer follow-up in subsequent studies. We include this important observation in the manuscript (lines 299-302).

- grammar and syntaxis must be thoroughly revised: MPV is often reported as VMP, numbers are reported with 1 to 3 decimals, the number is given with "." or ",".

**Response:**

We checked the grammar and syntaxis, the abbreviations are maintained as MPV, numbers reported with 2 decimals, and number are given with "," in all the manuscript.
